# Microstructure and Mechanical Properties of IN690 Ni-Based Alloy/316LN Stainless-Steel Dissimilar Ring Joint Welded by Inertia Friction Welding

**DOI:** 10.3390/ma17030695

**Published:** 2024-02-01

**Authors:** Yiqi Tong, Liangliang Zhang, Chao Li, Yueting Ma, Peng Li, Honggang Dong

**Affiliations:** School of Materials Science and Engineering, Dalian University of Technology, Dalian 116024, China; tongyiqi2021@163.com (Y.T.); zll15538029120@163.com (L.Z.); lichao2019@mail.dlut.edu.cn (C.L.); mayueting2012@163.com (Y.M.); donghg@dlut.edu.cn (H.D.)

**Keywords:** inertia friction welding, Inconel 690/316LN dissimilar joint, interfacial characteristics, impact toughness, fracture mechanism

## Abstract

Inertia friction welding (IFW) was used to join large-diameter hollow bars made of Inconel 690 and 316LN successfully. The interfacial characteristics, microstructure, mechanical properties and fracture mechanism of welded joints under different process parameters were investigated. The results indicated that a joining mechanism with mechanical interlocking and metallurgical bonding was found in IFW joints. There was a significant mechanical mixing zone at the welding interface. The elemental diffusion layer was found in the “wrinkles” of the mechanical mixing zone. A tiny quantity of C elements accumulated on the friction and secondary friction surfaces. The tensile strength and impact toughness of the joints increased with the total welding energy input. Increasing the friction pressure could make the grain in all parts of the joint uniformly refined, thus enhancing the mechanical properties of welded joints. The maximum tensile strength and impact toughness of the welded joint were 639 MPa and 146 J/cm^2^, reaching 94% and 68% of that for Inconel 690, respectively, when the flywheel was initially set at 760 rpm, 200 MPa for friction pressure, and 388 kg/m^2^ for rotary inertia. Due to the Kirkendall effect in the welded joint, superior metallurgical bonding was at the welding interface close to the Inconel 690 side compared to the 316LN side.

## 1. Introduction

The development of nuclear power is of great significance in ensuring energy supply and security, protecting the environment and optimizing the power industry structure [1]. In the nuclear power field, there are more Ni-base superalloy and stainless-steel dissimilar metal welding structures [2]. Therefore, the welding of stainless-steel and Ni-base superalloys has become a research hotspot. Inconel 690 alloy is a kind of solid solution-strengthening Ni-base superalloy with a face-centered cubic structure that has excellent heat and corrosion resistance. It has become the primary material for steam generator piping in pressurized water reactor nuclear power plants due to its low stacking fault, high thermal strength, outstanding metallurgical stability and excellent processing and manufacturing performance [3]. The main pipeline in a nuclear power plant connects the pressure vessel to the steam generator under severe working conditions. It must receive large loads and mechanical damage while in operation. Nitrogen-controlled austenitic stainless-steel 316LN has become the preferred material for the main pipeline at the first loop of a nuclear power plant, owing to its high-quality intergranular corrosion resistance, superior weldability and excellent tensile strength [4,5]. Therefore, the quality of the weld between the steam generator and the main pipeline directly determines the safety of the entire nuclear power plant.

The differences in physical and chemical properties between dissimilar materials can lead to solidification cracks, harmful phase precipitation, liquation cracks, insufficient mixing and other problems in fusion welded joints, thus deteriorating the quality of welded joints. Solidification cracks are a common problem in the fusion welding of Ni-base superalloy and austenitic stainless steel. The susceptibility of the solidification crack in the weld is related to the dilution rate of dissimilar metals. For welding between Ni-base superalloys and austenitic stainless steels, in general, the range of solidification temperatures for the weld metal increases with the dilution rate of austenitic stainless steels, which, in turn, increases the susceptibility of heterogeneous metal welds to solidification cracking [6,7,8]. Impurity elements such as S and P tend to form low-melting-point liquid films at grain boundaries, which can also increase the susceptibility to solidification cracking in the weld [9]. Haldar et al. [10] investigated the microplasma arc welding characteristics of Inconel 625 and 316L. They found the presence of a complex brittle phase, the Laves phase, formed at the joint due to Nb and Mo elemental segregation. The precipitation of a large number of brittle phases led to intercrystalline embrittlement, which reduced the toughness of the welded joints. Through the use of pulsed laser welding, Afshari et al. [11] investigated the weld characteristics of 4340 steel and the GTD-111 superalloy. They discovered liquation cracks in the heat-affected zone (HAZ) of the GTD-111 superalloy. Naffakh et al. [12,13] investigated the microstructure of welds between Inconel 657 and AISI 310 using shielded metal arc welding and gas tungsten arc welding. They found that defects such as the unmixed zone and the partially melted zone existed at the junction of the weld with the base metal. And there was a wide HAZ near the fusion line. Mahyari et al. [14] investigated the microstructure and mechanical properties of welds between T91 and T22 using gas tungsten arc welding. It was found that when ERNi-1 was selected as the welding wire, there was a distinctively sharp fusion boundary at the HAZ of T22 due to the presence of Ni. This resulted in increased hardness at the interface of the T22 steel and the weld metal. Compared to other wires, the strength of the joints made with ERNi-1 wire was reduced, but the elongation was significantly increased.

Since friction welding is a solid-state welding method, it avoids the common issues that arise in conventional fusion welding, such as element segregation, solidification cracks and liquefaction cracks. Due to its near-net-shape manufacturing aspect, high production efficiency and low cost of production, friction welding has been widely used worldwide [15]. Anitha et al. [16] investigated the influence of continuous drive friction welding (CDFW) on the mechanical characteristics and microstructure of Inconel 718/SS410 welded joints. It was discovered that raising the friction pressure and rotating speed could strengthen the tensile strength of joints. The effect of CDFW on the microstructure characteristics of welded joints between Inconel 718 and SM45C carbon steel was investigated by Murali et al. [17]. It was discovered that a fine-grained mechanical mixing zone developed at the welding interface. Zhu et al. [18] investigated the relationship between microstructure and mechanical properties of inertia friction welding (IFW) joints made of Inconel 751 and 21-4N. They found chemical mixing zones at the welding interface and the presence of carbides on the Inconel 751 side. The mechanical properties were demonstrated to be correlated with both the chemical mixing zone and the carbide layer close to the welding interface. Ding et al. [19] investigated the mechanical properties and microstructure of the IFW joint between K418 and 42CrMo. It was discovered that tensile fracture occurred on the K418 side, and that the formation and extension of the crack were associated with MC carbides. According to Luo et al. [20], the tensile strength of K418/42CrMo welded joints was enhanced by the features of current inertial friction welding (CIFW), including local occlusion, mechanical interlocking and diffusion bonding. Ajay et al. [21] investigated the mechanical properties of CDFW joints made of Inconel 718 and AISI 304. It was discovered that the tensile fracture was on the AISI 304 side, and a network of dimples was present on the fractured surface. Beeravolu et al. [22] investigated the effect of CDFW on the microstructure and mechanical properties of joints between IN718 and AISI 316L in both post-weld heat treatment (PWHT) and as-welded conditions. It was discovered that under both as-welded and PWHT conditions, the tensile fracture happened in the HAZ on the AISI 316L side.

As mentioned earlier, most of the early research focused on welding small-diameter bars made of Ni-base superalloys and stainless steels using CDFW. In this study, IFW was used to join large-diameter hollow bars made of Inconel 690 and 316LN. The interfacial characteristics, microstructure, mechanical properties and fracture mechanism of welded joints under different process parameters were investigated. The innovation of this study is the utilization of IFW technology instead of traditional fusion welding, which avoids common welding defects during the fusion welding of Ni-base superalloy and stainless steels in the production process and improves production efficiency. It is noteworthy that special welding wire and electrodes are not required, which significantly lowers costs and eases the process of welding when compared to the fusion welding currently employed in the nuclear power equipment manufacturing industry. The research results will enrich the basic theory of IFW and develop a new welding process for the nuclear power equipment manufacturing industry.

## 2. Materials and Methods

The solid solution-aged Ni-base superalloy Inconel 690 and the solid solution austenitic stainless-steel 316LN were employed as welding materials in this work. The chemical composition (wt.%) and physical properties are shown in Table 1 and Table 2, respectively. The welding workpieces were large-sized pipes with an outer diameter of 107 mm and an inner diameter of 70 mm. The surface of the workpiece was cleaned with alcohol before welding. Welding experiments were conducted using the HWI-IFW-130 axial/radial inertia friction welder. Different combinations of process parameters were designed to obtain IFW joints with excellent mechanical properties, as illustrated in Table 3. Samples #1, #2 and #3 were used to investigate the impact of initial rotating speed on the microstructure and mechanical properties of the joints. The impact of friction pressure on the joints was investigated for samples #3 and #4. The #5 sample was obtained by optimizing the parameters, and the specific optimization process is described in detail in Section 3.1.

**Figure 1:**
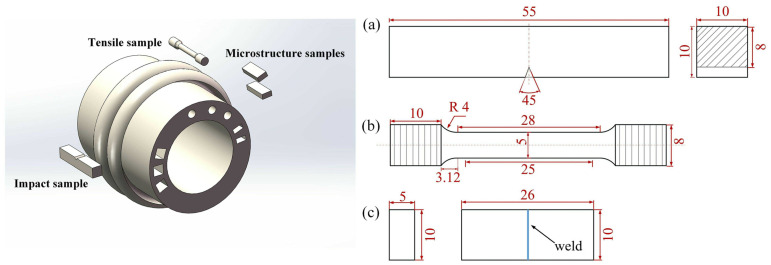
Sampling diagram and dimensions of samples for (**a**) impact, (**b**) tensile and (**c**) microstructure tests (unit: mm).

**Figure 2:**
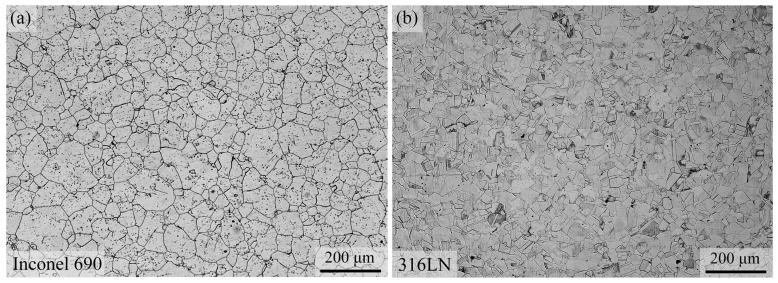
Microstructure of (**a**) Inconel 690 and (**b**) 316LN.

The rectangular samples of size 10 mm × 10 mm × 26 mm were taken from the center of the weld at a radial position, as shown in Figure 1. The samples were then mechanically ground and polished. The weld metal was electrolytically etched using a 10% oxalic acid solution at voltage 5 V, current 1 A and an etching time of 2 s. The metallographic samples of the two base materials were observed using an optical microscope (OM, LEICA DMi8, Leica Microsystems, Wetzlar, Germany). Both Inconel 690 and 316LN belong to the single-phase austenitic alloys, and their metallographic organizations are shown in Figure 2. The microstructure and element distributions of welded joints were conducted by high-resolution scanning electron microscopy (SEM, JSM-IT800 (SHL), Japan Electronics Co. Ltd., Tokyo, Japan) with an energy-dispersive spectrometer (EDS) and an electron probe micro-analyzer (EPMA, JXA-8530F Plus, Japan Electronics Co. Ltd, Tokyo, Japan) with a wave-dispersion spectrometer (WDS), respectively. The grain orientation and grain size of the IFW joint were determined by electron backscatter diffraction (EBSD, JSM-IT800 (SHL), Japan Electronics Co. Ltd, Tokyo, Japan). The observed surface was polished with a vibration polishing machine (VibroMet2, Buehler Ltd, Lake Bluff, America) to effectively remove the strained layer, and the vibration polishing time was about 10 h.

The tensile test was conducted on an Instron 5982 (Instron, MA, USA) universal test machine with a crosshead speed of 5 mm/min. The JBW-300B (Jinan Time Shijin Instrument Co., Ltd., Jinan, China) instrumented impact tester was used for the impact test of joints under different process parameters. For every processing state, three samples were prepared, and the test result was determined by taking the average value. The dimensions and sampling positions of tensile and impact samples are shown in Figure 1. The fracture morphology and chemical composition of the fracture surface were analyzed by scanning electron microscopy (Zeiss SUPRA55, Carl Zeiss AG, Oberkochen, Germany) with EDS.

## 3. Results and Discussion

### 3.1. Morphology of the Welded Joints

**Figure 3:**
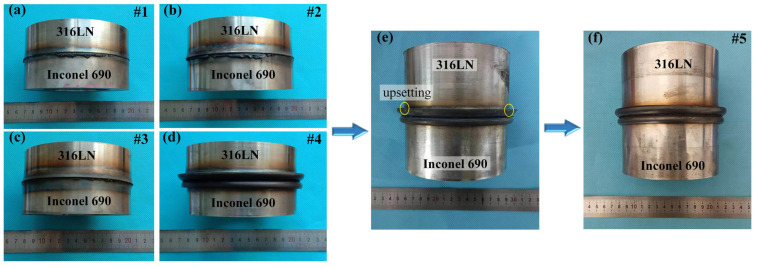
Appearance of the IFW joints (**a**) #1, (**b**) #2, (**c**) #3, (**d**) #4, (**e**) with friction pressure increased to 200MP based on #4 joint and (**f**) #5. The upsetting phenomenon was marked in (**e**) with the yellow circle.

The macroscopic morphology of the IFW joints with different welding parameters is shown in Figure 3. The upper and lower parts of the joint were 316LN and Inconel 690, respectively. It can be found that the flash of the joint was mainly formed on the 316LN side. This is due to the lower thermal strength of 316LN compared to Inconel 690, which allows for more plastic deformation on the 316LN side at welding temperatures.

It can be found from the IFW joints #1, #2 and #3 that the size of the flash increased with the initial speed of the flywheel. This is because the total welding energy input increased with the initial speed of the flywheel. The reduction in material strength at high temperatures caused more plastic deformation of the joint, and the high-temperature metal was extruded to form a flash under the influence of axial pressure. The relationship between the stored energy *E* (J), the rotary inertia *I* (kg/m^2^) and the initial speed *ꞷ* (rad/s) of the flywheel for IFW is as follows [23]:

(1) $$E=\frac{1}{2}I\omega^{2}$$

(2) $$I=\frac{1}{2g}GR^{2}$$

where *G* is the flywheel gravity (N), *R* is the flywheel radius (m) and *g* is the gravitational constant (9.8 N/kg).

It can be found from the IFW joints #3 and #4 that the size of the flash increased with the friction pressure. This is because increasing the friction pressure could promote the extrusion of oxidized metal and other harmful impurities in the joints to form the flash. This process would help to forge the metal in the joint and refine the grains, improving the mechanical properties of the joint [15]. It is worth noting that the flash on both sides of the #4 joint did not completely coil, indicating that the friction pressure of 170 MPa was not sufficient to form a friction-welded joint with a favorable flash shape.

When the friction pressure increased to 200 MPa, it was found that the flash of the stainless steel was not completely coiled, and the upsetting phenomenon was found at the root of the flash, as shown in Figure 3e. It indicated that there was not enough total welding energy. Consequently, the initial speed and rotary inertia of the flywheel should be adjusted to increase the total energy input. It can be seen from Equation (1) that the initial speed of the flywheel has a greater influence on the total energy input than the rotary inertia. The excessively high rotating speed will lead to the coarsening of the grain at the joint, affecting the mechanical properties of the joint [24]. Therefore, based on the #4 joint, rotary inertia and friction pressure were increased, the initial speed was reduced and then the #5 joint was obtained. It was observed that the flash shape of the #5 joint was excellent, indicating that sufficient plastic deformation had occurred in the metal on both sides of the weld.

### 3.2. Microstructure at the Interface Zone

**Figure 4:**
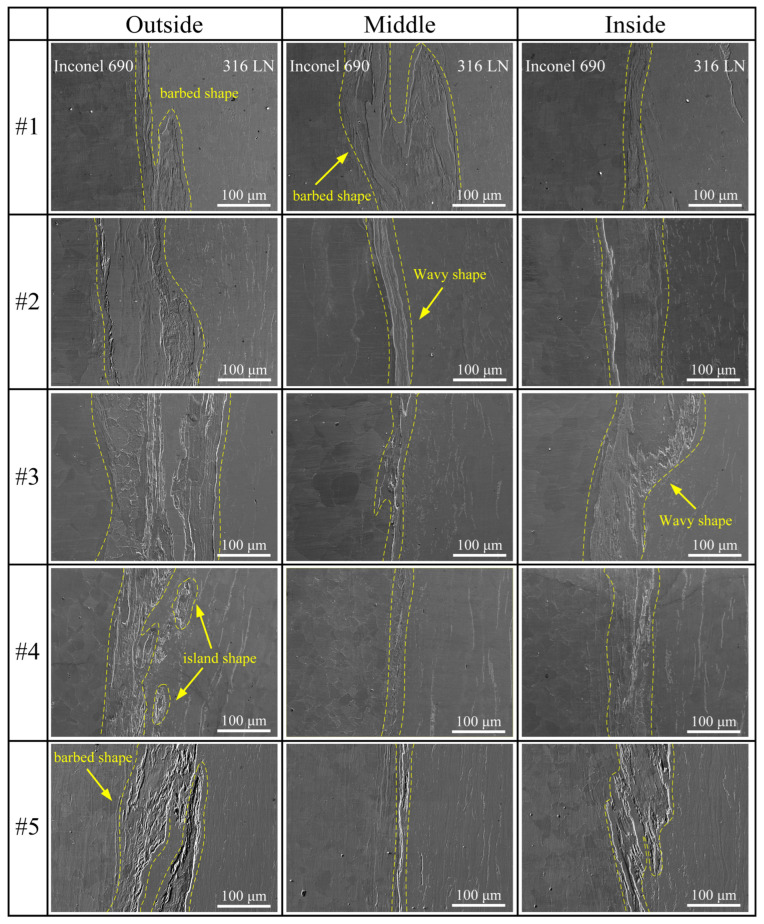
Microstructure of the interfaces around the middle, outside and inside of the joints.

Throughout the friction welding process, strain hardening, plastic rheology, mechanical interlocking, dynamic recrystallization and diffusion of alloying elements occurred at the friction interface because of the repeated cycles of thermo-mechanical coupling [16,17,20]. These factors will impact the welding quality of joints, which impacts their mechanical properties. The SEM images of the welding interface are displayed in Figure 4. The welding interface was wavy, barbed and island-shaped. It is worth noting that the Ni-base superalloy was inserted into the stainless-steel side at the interface. This is because under the effect of axial pressure and friction torque, the micro-convexities on the surface of Ni-base superalloy with higher thermal strength would be pressed into the softer stainless-steel surface and metallurgically bonded under the action of thermo-mechanical coupling, thus generating secondary friction surfaces. Such an inlay structure could play the role of mechanical interlocking and, at the same time, contribute to the addition of the contact area of the welding interface, which, in turn, promotes the diffusion of elements in the interface area.

It was observed that the welding interface width in the middle region of the #1 joint was greater than that of the inside and the outside, while the opposite was true for the other samples. For the IFW of pipes, the middle region of the interface is the first to heat up at the beginning of the welding process, with a tendency toward high temperatures at the center and low temperatures at the edges [25]. Because the initial speed and rotary inertia of the #1 joint were minor, the welding time was insufficient, and there was no complete heat generation process compared with other samples; the temperature in the middle region of the weld was higher at the end of welding. This caused intense mechanical mixing and elemental diffusion to occur in the middle of the weld compared to the sides. In normal circumstances, owing to more severe plastic deformation on the inside and outside during the welding process, which encouraged mechanical occlusion and metallurgical bonding, other joints showed a wider welding interface on both sides.

From IFW joints #1, #2 and #3, as the initial speed increased, the welding interface width narrowed in the middle of the joints and grew wider on both sides. This is because the welding energy input is positively correlated with the initial speed, and the increase in energy input promotes the plastic deformation of the weld metal. Under axial pressure, the softened material in the middle of the joint moved to the sides, resulting in a change in the radial welding interface width. From IFW joints #3, #4 and #5, the increase in friction pressure promoted the extrusion of the plastic metal on both sides of the joint in the form of a flash, resulting in a decrease in the width of the welding interface on both sides with the increase in friction pressure.

**Figure 5:**
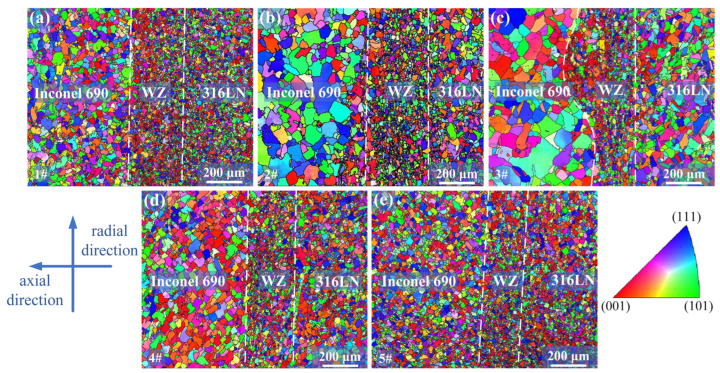
EBSD results of joints (**a**) #1, (**b**) #2, (**c**) #3, (**d**) #4 and (**e**) #5.

The microstructure characteristics of IFW joints under different parameters were analyzed by EBSD technology. As illustrated in Figure 5, a significant orientation difference between neighboring grains was visible on the IPF map. It indicated that new equiaxed grains were generated in the weld zone (WZ) by dynamic recrystallization under the thermo-mechanical coupling [26,27]. During the welding process, there was significant plastic deformation in the WZ, which produced deformed grains with a high dislocation density. At the same time, because the peak temperature at the weld was higher, it provided conditions for the dynamic recrystallization of deformed grains [21]. Significant grain refinement was found in the WZ. This is because the short welding time and rapid cooling rate of IFW meant that the recrystallized grains did not grow sufficiently, resulting in the formation of a fine-grain structure at the weld. Han et al. [28] found that in rolled 316L plates, the texture led to differences in the mechanical properties of the material in different directions. As shown in Figure 5, a fine-grain zone existed in the WZ, and there was a clear orientation difference between these grains. It indicated that the microstructure of the friction welding joints was isotropic, which avoided differences in properties in the radial and axial directions.

**Figure 6:**
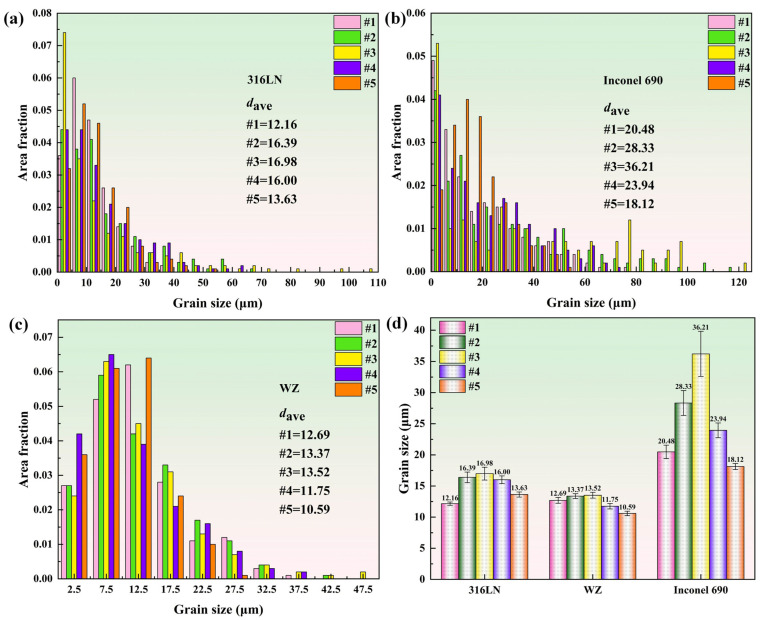
Grain size distributions in (**a**) 316LN side, (**b**) WZ and (**c**) Inconel 690 side of joints; (**d**) statistical results of grain size under different welding parameters.

The grain sizes of the 316LN side, WZ and Inconel 690 side of the joints under different process parameters were counted, respectively, and the results are presented in Figure 6. The #3 joint had the largest average grain size in each part, with 16.98 μm, 13.52 μm and 36.21 μm, respectively. This is because the welding energy input increased with the initial speed and the welding time was prolonged, which led to grain coarsening. The average grain size of the #5 joint was minor with 13.63 μm, 10.59 μm and 18.12 μm, respectively. By increasing the friction pressure, the microstructure of the joint could be sufficiently forged, plastic deformation could be promoted and the welding time could be shortened [29]. All these factors contributed to the reduction in grain size. It can be seen from Figure 3 that during the welding process, the flash size of the #1 sample was the minimum; that is, the plastic deformation generated during the welding process was the minimum. The metal with dynamic recrystallization close to the friction surface of other samples was continuously extruded in the form of flash, and the plastic deformation increased with adjustments in process parameters. This resulted in the continuous generation of new recrystallized grains in the WZ for other samples. In the #1 sample, because of the small amount of plastic deformation, most of the metal that dynamic recrystallization close to the friction surface remained in the WZ. For the #1 sample, although the welding time was short and the heat generation process was incomplete, there was a relatively long residence time for the recrystallized grains in the WZ. This resulted in relatively larger grain size in the WZ compared to the 316LN side for the #1 sample. The larger size of the welding interface in the middle region of the #1 sample in Figure 4 also indicated that most of the metal that underwent dynamic recrystallization close to the friction surface remained in the WZ. It is worth noting that Inconel 690 exhibited more noticeable grain coarsening at the weld compared to 316LN. Because there was a lower thermal conductivity in Inconel 690 than in 316LN, the temperature of the Inconel 690 side was higher during welding than the 316LN side. The higher temperature on the Inconel 690 side contributed to grain growth. It was also discovered that the difference in grain size in each part of the #5 joint was the least significant.

### 3.3. Interface Formation Mechanism

**Figure 7:**
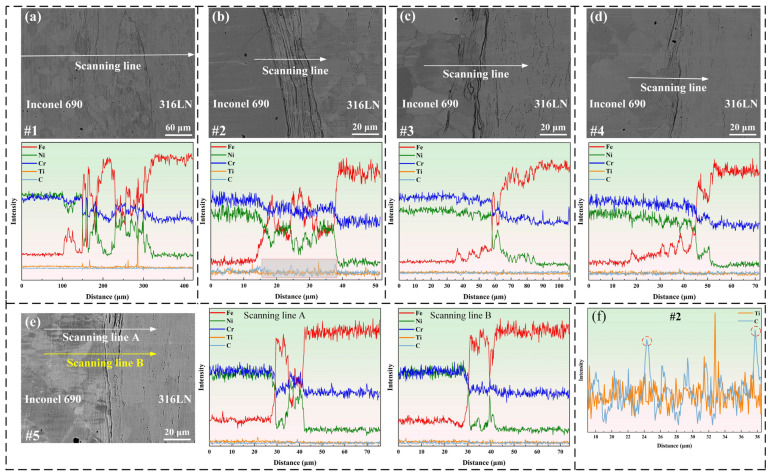
Microstructure and EDS line scan analyses across the interface of joints (**a**) #1, (**b**) #2, (**c**) #3, (**d**) #4 and (**e**) #5; (**f**) high magnification of (**b**).

The welding interface morphology and EDS scanning results at the middle region of joints are shown in Figure 7. The findings indicated that the appearance and size of the welding interface were varied at different process parameters. The solid-state diffusion of major elements occurred under thermo-mechanical coupling. The fluctuation in the intensity of the elements occurred in the welding interface, indicating that the welding interface was a cross-distributed layered structure of Inconel 690 and 316LN. The layered structure consisted of the friction surface and the secondary friction surface. The distribution of C elements at the welding interface was investigated in the #2 joint as an example, as illustrated in Figure 7f. Agglomeration of C elements was discovered in the interlayer, and other joints showed similar conditions. It is worth noting that the diffusion distance of the Fe elements on the Inconel 690 side was larger than that of the Ni elements on the 316LN side.

**Figure 8:**
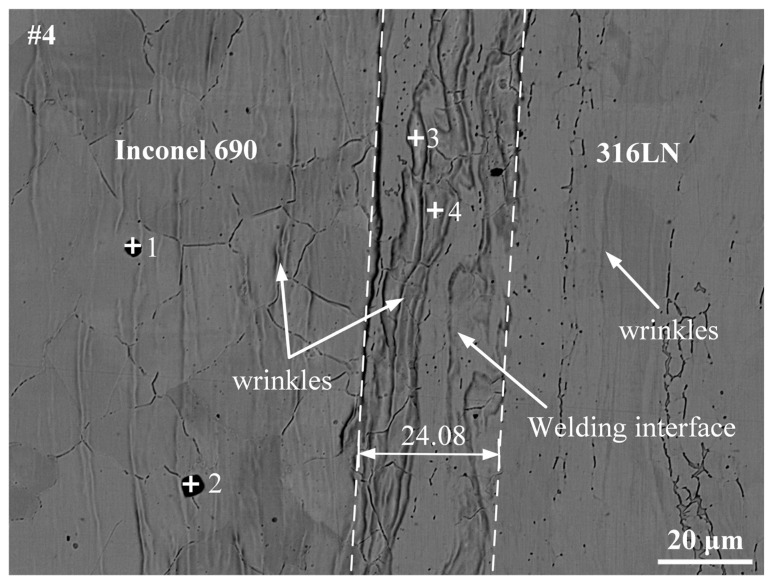
Interfacial microstructure at #4 joint.

The #4 joint was used as an example to further analyze the metallurgical bonding at the welding interface. As shown in Figure 8, in addition to the welding interface, there were “wrinkles” perpendicular to the direction of friction pressure on both sides of the base metal in the joint. The width at the welding interface of the #4 joint was about 24.08 μm. The EDS was used to examine the phase of the Inconel 690 side and the composition at various locations of the welding interface. The +1, +2, +3 and +4 marked in Figure 8 are the locations of the spots in which the chemical composition was examined. The results are displayed in Table 4. The phase on the Inconel 690 side was determined to be Ti(C, N), and Rehman et al. [30] also found TiN particles in Inconel 600. Because MC and MN have the same lattice type with N and C atoms substituting for each other, there was an M(C, N)-type carbonitride on the Inconel 690 side. It has been demonstrated that Ti(C, N) does not adversely affect the mechanical properties of friction-welded joints but plays a “pinning effect” on grain boundaries and is conducive to promoting grain refinement [15]. The EDS results revealed that the “wrinkles” in the welding interface were mainly solid solutions of Fe, Cr and Ni. This indicated that sufficient diffusion of the element had occurred at the “wrinkles”.

**Figure 9:**
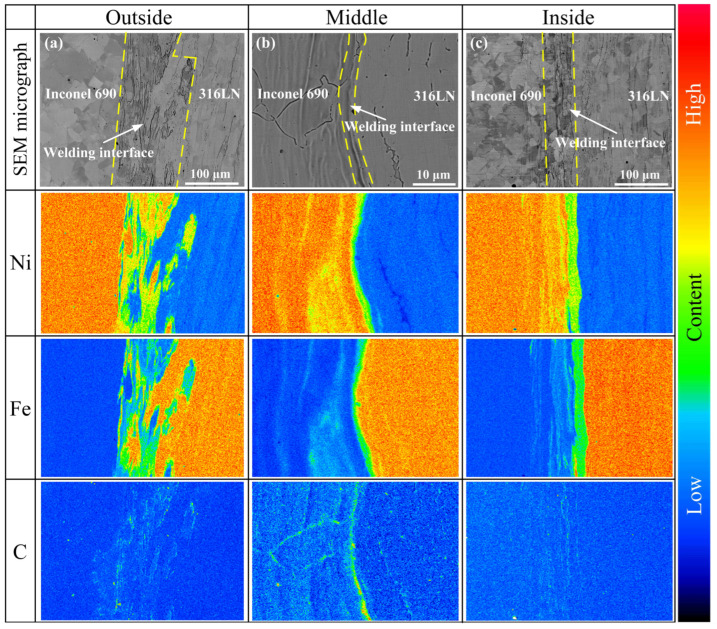
Elemental distribution of the across the interfaces on the (**a**) outside, (**b**) middle and (**c**) inside regions of the #4 joint.

**Figure 10:**
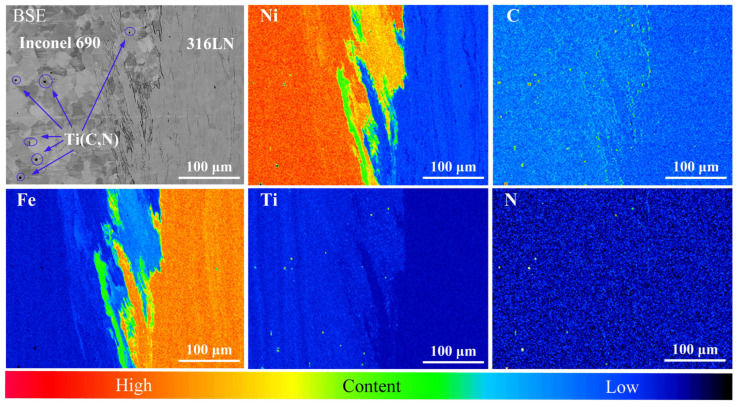
Elemental distribution across the interfaces around the inside of the #5 joint.

The EPMA was used to further determine the element distribution at the welding interface on the outside, inside and middle regions of the #4 joint. There were elemental diffusion layers at the welding interface, as shown in Figure 9. Furthermore, these diffusion layers were mainly concentrated in the “wrinkles”. The formation of these “wrinkles” could be connected to both axial frictional pressure and the diffusion of elements. It is noteworthy that the aggregation of the C elements was found at the periphery of the welding interface. The periphery of the welding interface happened to be the friction and secondary friction surfaces, and the high peak temperatures at these positions during the welding process promoted elemental diffusion. Simultaneously, the interfacial energy was high due to the significant lattice distortion at the welding interface, which furthered the enrichment of C elements at the interface [31]. The aggregation of the C elements at the friction surface of the #5 joint was less pronounced than that of the #4 joint, as illustrated in Figure 10. It could be because increasing the friction pressure and rotary inertia promoted the uniform distribution of C elements in the vicinity of the friction surface. Ding et al. [19] also discovered the carbide layer on the K418 side of the IFW joint between 42CrMo and the K418 alloy. Ti(C, N) particles were also discovered on the Inconel 690 side of the #5 joint. It is worth noting that significant diffusion of Fe elements was found on the Inconel 690 side close to the welding interface in both joints #4 and #5. This is due to the occurrence of the Kirkendall effect in the welded joint. In other words, the diffusion rate of Fe in Ni was greater than that of Ni in Fe, which led to unequal diffusion at the welding interface [32]. It can affect the metallurgical bonding on both sides of the welding interface and then impact the mechanical properties of the joint.

### 3.4. The Mechanical Properties of the Joints

#### 3.4.1. Tensile Strength

**Figure 11:**
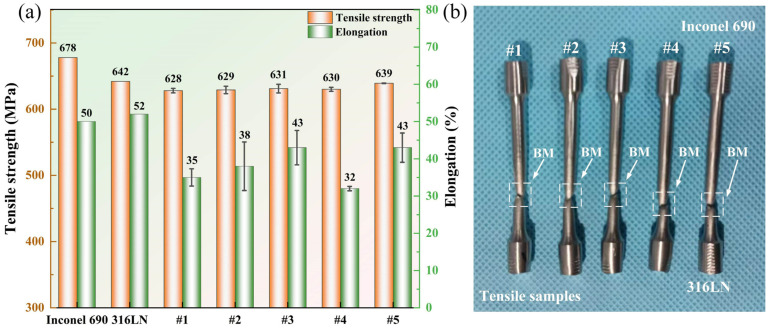
(**a**) Tensile strength and elongation of the IFW joints, and (**b**) photographs of the fracture locations for samples prepared at different welding parameters.

The tensile strength results of Inconel 690/316LN IFW joints under different process parameters indicated that in the case of constant pressure and rotary inertia, the effect of increasing the initial speed on the tensile strength was limited. The tensile strengths of joints #1 to #3 were maintained at the same level, and the tensile strength of the #3 joint was 631 MPa, reaching 98% and 93% of that for 316LN and Inconel 690, respectively. It is worth noting that there was a significant effect on elongation by increasing the initial speed. The elongation increased from 35% to 43%, reaching 86% of the base metal. This is because the peak temperature at the welding interface and the welding energy input increased with the initial speed, and the welding time was prolonged. This can encourage the plastic flow of the two materials as well as metallurgical bonding at the welding interface, which, in turn, would increase the elongation of the joint. The reduction in elongation of the #4 joint may be due to the serious strain hardening at the joint caused by the increase in friction pressure. The deformation of the material at the weld during the tension process was made more difficult due to the stacking of dislocations. After process optimization, the tensile strength of the #5 joint was 639 MPa, or roughly 94% and 100% of Inconel 690 and 316LN, respectively, with a 43% elongation. On the one hand, the rise in rotary inertia of the #5 joint increased the welding energy input, and, on the other, it extended the welding time. All these factors were favorable to improving the conversion efficiency of welding energy input [33], resulting in adequate dynamic recrystallization at the welding interface. It can be seen from Figure 5 and Figure 6 that the grains in each region of the #5 joint were uniformly refined. The refined grain prevented dislocation movement so that dislocations could only be stacked at grain boundaries and reduced the speed of movement, thus strengthening the tensile strength of the joint [34]. In the #5 joint, not only did obvious grain refinement occur but the difference in grain size in each part was the least significant. As a result, the plastic deformation was uniformly distributed in several grains during the stretching process. Therefore, the number of dislocations accumulated at the grain boundaries was reduced, which would be conducive to strain compatibility between the grains, to avoid premature crack initiation, resulting in material fracture. These factors contributed to the increased elongation of the #5 joint [35]. The tensile strength and elongation of the #5 joint could be improved by sufficient dynamic recrystallization and fine-grain strengthening. This strengthening satisfied the Hall–Petch relationship. The macroscopic fracture positions of the welded joints with different process parameters are shown in Figure 11b. The fracture positions were all located at the 316LN base metal, indicating excellent bonding at the welding interface.

#### 3.4.2. Impact Toughness

**Figure 12:**
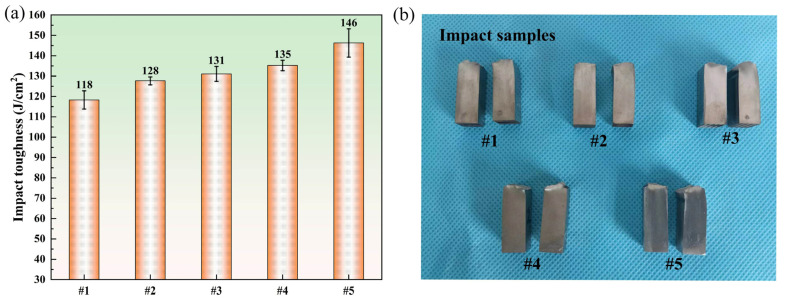
(**a**) Charpy impact toughness of the IFW joints, and (**b**) photographs of the fracture locations for samples prepared under different welding parameters.

The impact toughness results of Inconel 690/316LN dissimilar metal IFW joints with different process parameters are shown in Figure 12. The impact toughness of the #5 joint was the maximum, at 146 J/cm^2^, reaching 68% of that for Inconel 690. The impact toughness of the #1 joint was the minimum, at 118 J/cm^2^, reaching 55% of that for Inconel 690. It has been established that the welding time is the decisive factor influencing the impact toughness of friction-welded joints [36]. This is because an excessively short welding time means an extremely low energy input, which is not enough to produce sufficient metallurgical bonding at the joint. However, excessively long welding times could lead to grain coarsening at the joint, which, in turn, affects the mechanical properties of the welded joints. It can be seen that the impact toughness of the joints increased with the initial speed from Figure 12, which was related to the improvement in the welding energy input.

The smaller the grain size was, the greater the number of grains that existed at the joint. There was more uniform plastic deformation and less stress concentration since the plastic deformation could be distributed over a larger number of grains. In addition, the finer the grain was, the larger the area of the grain boundary was, and the more tortuous the grain boundary was, which hindered crack propagation. These factors were the main reasons for the improvement in the impact toughness of joints #4 and #5. At the same time, the improvement in the impact toughness of the #5 joint was also due to the reasonable matching of the welding energy input with the plastic deformation at the joint. For the #5 joint, the difference in grain size in each part was the minimum, and the microstructure was more uniform. It avoided the crack extending to the coarse-grained area on both sides of the weld in the process of ductile fracture.

### 3.5. Analysis of Fracture Surfaces

#### 3.5.1. Fracture Morphology of the Tensile Samples

**Figure 13:**
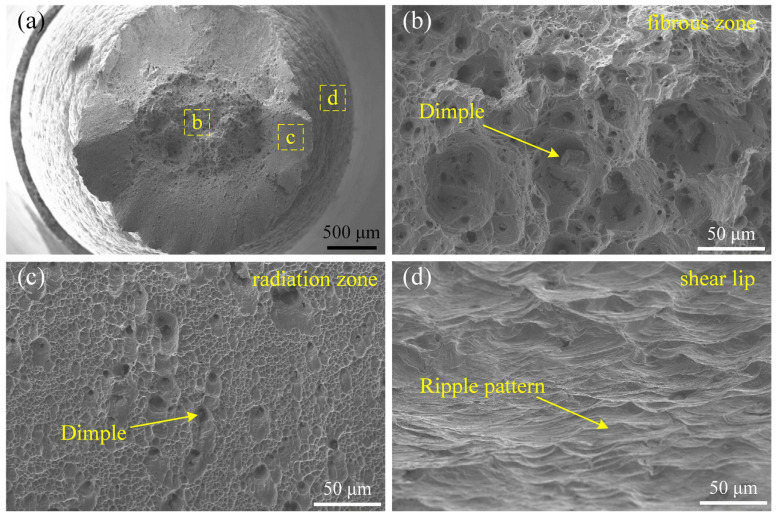
Morphology of the tensile fracture surface of #5 joint. (**a**) Macro morphology; (**b**–**d**) high magnification of yellow dashed box in (**a**).

Since the tensile properties and fracture morphology of the joints were similar under different process parameters, the #5 joint, as an example, was observed in the microstructure of the tensile fracture surface by SEM. It is visible in Figure 13 that there was a ductile fracture at the joint, and the fracture appearance was a typical cup cone. From the inside to the outside, fractured surfaces could be divided into three regions: the fibrous zone, the radiation zone and the shear lip. A large number of equiaxed dimples with different sizes and depths were found in the fibrous zone. Compared with the fibrous zone, the dimples in the radiation zone were smaller and denser. In addition, a typical ripple pattern was found in the shear lip.

#### 3.5.2. Fracture Morphology of the Impact Samples

**Figure 14:**
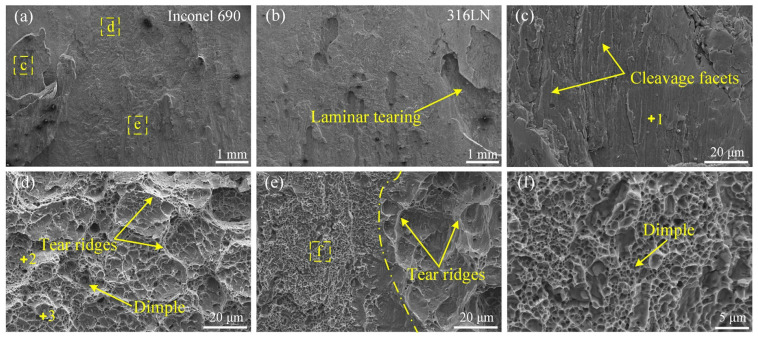
Morphology of the impact fracture surface of #1 joint. (**a**,**b**) Macro morphology; (**c**–**e**) high magnification of yellow dashed box in (**a**); (**f**) high magnification of yellow dashed box in (**e**).

**Figure 15:**
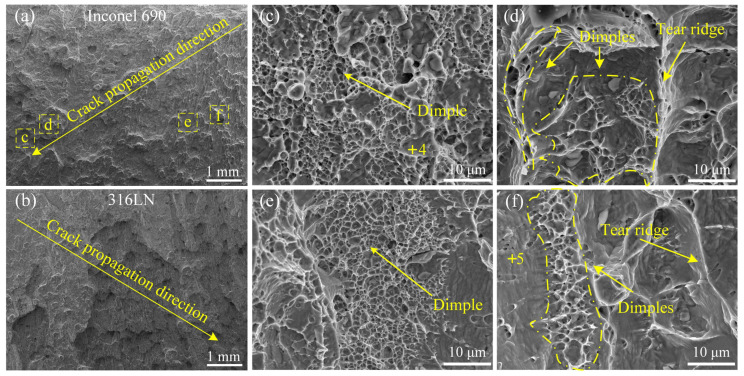
Morphology of the impact fracture surface of #3 joint. (**a**,**b**) Macro morphology; (**c**–**f**) high magnification of yellow dashed box in (**a**).

**Figure 16:**
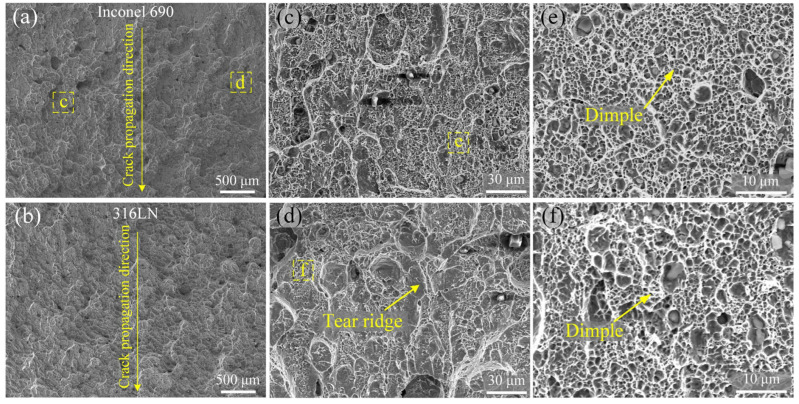
Morphology of the impact fracture surface of #5 joint. (**a**,**b**) Macro morphology; (**c**,**d**) high magnification of yellow dashed box in (**a**); (**e**,**f**) high magnification of yellow dashed box in (**c**,**d**).

The impact fracture morphology was observed using SEM, as shown in Figure 14, Figure 15 and Figure 16. Considering the impact toughness and fracture morphology characteristics of different welded joints, joints #1, #3 and #5 were selected as examples for observation in this study. The upper sides of Figure 14a,b, Figure 15a,b and Figure 16a,b were near the notched position of the impact sample. 

High-magnification images of the yellow boxed regions “c”, “d” and “e” in Figure 14a are displayed in Figure 14c,d,e, respectively. There were randomly distributed protrusions on the fracture surface of Inconel 690, while on the 316L side, it was the opposite. It can be seen from Figure 14a,b that there was typical laminar tearing at the impact fracture of the #1 joint. The laminar tearing on the Inconel 690 side was magnified and observed, as shown in Figure 14c. It can be noticed that there were cleavage facets on the surface, showing the characteristics of a brittle fracture. More equiaxed dimples can be observed with a few tear ridges around them in Figure 14d. Small and dense dimples are distributed on the left side of Figure 14e, and a large number of tear ridges are gathered on the right side. The phenomenon of laminar tearing at the fracture of the #1 joint indicated that the connection between the two metals at individual positions of the weld relied mainly on macroscopic mechanical occlusion. This caused the joint to tear and fail under the action of an impact force.

The composition at different positions of the #1 joint was analyzed using EDS, and the results are shown in Table 5. The +1, +2 and +3 marked in Figure 14 are the locations of the spots in which the chemical composition was examined. The analysis indicated that there were Ti(C, N) particles at the bottom of the dimples. The Ni content of spot 2 was higher than that of spot 1, while the Fe content was lower than that of spot 1, meaning that spot 2 was closer to Inconel 690 than spot 1.

The impact fracture morphology of the #3 joint is illustrated in Figure 15. The high-magnification images of the yellow boxed regions “c”, “d”, “e” and “f” in Figure 15a are displayed in Figure 15c,d,e,f, respectively. It was established that the crack originated on the inside of the joint and expanded towards the outside. It indicated that the metallurgical bonding on the outside of the joint was more sufficient. There were many protrusions on the fracture surface of the Inconel 690 side, and the size of the protrusions increased gradually along the crack propagation direction. In Figure 15a, “d” and “f” are located at the large-size protrusion and the small-size protrusion, respectively. And “c” and “e” were located at the pits around the large-size protrusion and the small-size protrusion, respectively. Numerous equiaxed dimples of varying sizes and depths were dispersed at the pits surrounding the protrusions, as shown in Figure 15c,e. As can be seen from Figure 15d,f, there was a mixed morphology with dimples and tear ridges at the protrusions. This illustrated that the metallurgical bonding was more sufficient at the pit of the Inconel 690 side than in the protrusion [37]. The #3 joint exhibited a mixed fracture with a quasi-dissociative and ductile nature. A similar phenomenon also occurred at the #1 joint. From Figure 14, “d” was closer to Inconel 690 than “c” and “e”, and the number of dimples in “d” was higher. However, “c” was closer to 316LN and exhibited brittle fracture characteristics.

The composition at different positions of the #3 joint was analyzed using EDS, and the results are shown in Table 6. The +4 and +5 marked in Figure 15 are the locations of the spots in which the chemical composition was examined. The Ni content of spot 4 was higher than that of spot 5, while the Fe content was lower than that of spot 5, meaning that spot 4 was closer to Inconel 690 than spot 5. It is noteworthy that the number of dimples was relatively high at spots 2 and 4, whereas the number of tear ridges and cleavage faces at spots 1 and 5 was relatively high. By inference, it can be seen that the metallurgical bonding was more efficient at the welding interface close to the Inconel 690 side. This is because the welding interface close to the Inconel 690 side had more adequate atom diffusion due to the Kirkendall effect at the joint.

The impact fracture morphology of the #5 joint is illustrated in Figure 16. Observing Figure 16a,b, it can be determined that the crack originated at the notch position and extended to the other side. This indicated that the metallurgical binding of both the inside and the outside at the joint was excellent. Compared to joints #1 and #3, the fracture surface of the Inconel 690 side at the #5 joint was distributed with protrusions of similar size. The high-magnification images of the yellow boxed regions “c” and “d” in Figure 16a are displayed in Figure 16c,d. The “d” and “c” were located at protrusions and pits, respectively. The high-magnification images of the yellow boxed regions “e” and “f” in Figure 16c,d are displayed in Figure 16e,f. It is worth noting that there were a large number of dimples in both the pits and the protrusions of the #5 joint, but there were a few tear ridges in the protrusions. In summary, the #5 joint exhibited a typical ductile fracture dominated by dimples.

## 4. Conclusions

In this work, inertial friction welding (IFW) was successfully used to join large-diameter hollow bars made of Ni-base superalloy Inconel 690 and austenitic stainless-steel 316LN. The tensile strength, impact toughness and microstructure characteristics of friction-welded joints under different process parameters were researched. This study could be summarized with the following conclusions:

(1) Metallurgical bonding and mechanical interlocking were employed as the joining mechanisms in IFW joints. A significant mechanical mixing zone was present at the welding interface. There was an element diffusion layer in the “wrinkles” of the mechanical mixing zone. A tiny quantity of C element aggregation existed at the friction and secondary friction surfaces.
(2) The grain size of the joint increased with the initial speed of the flywheel. Increasing the friction pressure could improve the mechanical properties of welded joints by uniformly refining the grain throughout the joint.
(3) When the flywheel was initially set at 760 rpm, 200 MPa for friction pressure and 388 kg/m^2^ for rotary inertia, the welded joint could simultaneously obtain the maximum tensile strength of 639 MPa and elongation of 43%, reaching 94% and 86% of that for Inconel 690, respectively. All the tensile samples exhibited ductile fractures.
(4) The impact sample of the #5 joint exhibited a ductile fracture. The maximum impact toughness was 146 J/cm^2^, which was 68% of that for Inconel 690. It was evident that the metallurgical bonding at the welding interface close to the Inconel 690 side was superior to that close to the 316LN side due to the Kirkendall effect at the joint.

## Figures and Tables

**Table 1:** Chemical composition (wt.%) of base metals.

Material	Ni	Fe	Cr	Ti	Mn	Mo	Si	N	C
Inconel 690	60.61	8.85	29.35	0.28	0.38	0.05	0.24	0.03	0.03
316LN	11.45	67.11	17.57	-	1.23	2.09	0.42	0.12	0.01

**Table 2:** Physical properties of base metals.

Material	Tensile Strength (MPa)	Melting Point (°C)	Elongation (%)	Impact Toughness (J/cm^2^)	Thermal Conductivity (W/(m·K))
Inconel 690	678	1343~1377	50	216	13.5
316LN	643	1400	55	350	15.0

**Table 3:** Process parameters of IFW.

Trial No.	Initial Rotating Speed (rpm)	Friction Pressure (MPa)	Rotary Inertia (kg·m^2^)	Initial Flywheel Kinetic Energy (kJ)
#1	650	70	280	647.1
#2	750	70	280	861.5
#3	850	70	280	1106.6
#4	850	170	280	1106.6
#5	760	200	388	1225.9

**Table 4:** EDS results (at.%) of the spots marked in Figure 8.

Spots	Ni	Fe	Cr	Ti	C	N	Al	Si	Mn	Co	Mo	Possible Phase
1	15.39	0.49	2.17	41.67	8.33	29.52	1.06	0.17	0.03	0.07	1.10	Ti(C, N)
2	15.09	3.46	9.94	36.47	9.82	24.37	0.49	0.24	0.09	0.03	-	Ti(C, N)
3	48.91	19.10	28.97	0.35	0.27	0.39	0.60	0.41	0.82	0.01	0.18	(Fe, Ni, Cr)_ss_
4	45.93	22.20	27.35	0.18	1.09	0.94	0.64	0.58	0.74	0.03	0.31	(Fe, Ni, Cr)_ss_

**Table 5:** EDS results (at.%) of the spots marked in Figure 14.

Spots	Ni	Fe	Cr	Ti	Mn	Co	Mo	Nb	Possible Phase
1	9.92	64.69	21.03	0.27	1.55	0.44	1.96	0.14	(Fe, Ni, Cr)_ss_
2	29.07	41.47	26.09	0.27	1.88	0.06	1.16	-	(Fe, Ni, Cr)_ss_
3	2.38	2.14	2.93	91.57	0.21	0.14	0.27	0.36	Ti(C, N)

**Table 6:** EDS results (at.%) of the spots marked in Figure 15.

Spots	Ni	Fe	Cr	Ti	Mn	Co	Mo	Nb	Possible Phase
4	58.62	7.39	32.20	0.54	0.87	-	0.32	0.06	(Fe, Ni, Cr)_ss_
5	11.90	63.79	19.35	0.10	2.60	0.75	1.51	-	(Fe, Ni, Cr)_ss_

## Data Availability

Data are contained within the article.
